# The Anatomical Boundary of the Rat Claustrum

**DOI:** 10.3389/fnana.2019.00053

**Published:** 2019-05-31

**Authors:** Christopher M. Dillingham, Mathias L. Mathiasen, Bethany E. Frost, Marie A. C. Lambert, Emma J. Bubb, Maciej M. Jankowski, John P. Aggleton, Shane M. O’Mara

**Affiliations:** ^1^Institute of Neuroscience, Trinity College Dublin, Dublin, Ireland; ^2^School of Psychology, Cardiff University, Cardiff, United Kingdom; ^3^Faculty of Basic and Applied Sciences, University of Poitiers, Poitiers, France; ^4^Department of Neurobiology, The Hebrew University of Jerusalem, Jerusalem, Israel

**Keywords:** parvalbumin, crystallin mu, Gng2, midline thalamus, neuroanatomical tracing, immunohistochemistry, immunofluorescence

## Abstract

The claustrum is a subcortical nucleus that exhibits dense connectivity across the neocortex. Considerable recent progress has been made in establishing its genetic and anatomical characteristics, however, a core, contentious issue that regularly presents in the literature pertains to the rostral extent of its anatomical boundary. The present study addresses this issue in the rat brain. Using a combination of immunohistochemistry and neuroanatomical tract tracing, we have examined the expression profiles of several genes that have previously been identified as exhibiting a differential expression profile in the claustrum relative to the surrounding cortex. The expression profiles of parvalbumin (PV), crystallin mu (Crym), and guanine nucleotide binding protein (G protein), gamma 2 (Gng2) were assessed immunohistochemically alongside, or in combination with cortical anterograde, or retrograde tracer injections. Retrograde tracer injections into various thalamic nuclei were used to further establish the rostral border of the claustrum. Expression of all three markers delineated a nuclear boundary that extended considerably (∼500 μm) beyond the anterior horn of the neostriatum. Cortical retrograde and anterograde tracer injections, respectively, revealed distributions of cortically-projecting claustral neurons and cortical efferent inputs to the claustrum that overlapped with the gene marker-derived claustrum boundary. Finally, retrograde tracer injections into the thalamus revealed insular cortico-thalamic projections encapsulating a claustral area with strongly diminished cell label, that extended rostral to the striatum.

## Introduction

The claustrum is a highly conserved nucleus that is not only present in all placental species (Baizer et al., 2014) but is also found in *Aves* (Puelles et al., 2016). The claustrum also exhibits genetic characteristics (Mathur et al., 2009; Smith and Alloway, 2010; Pirone et al., 2012; Hinova-Palova et al., 2014a,b; Kim et al., 2016), and cortical connectivity (Smith and Alloway, 2010; Patzke et al., 2014; Smith et al., 2014; Kitanishi and Matsuo, 2016; Wang et al., 2017; White et al., 2017; Qadir et al., 2018; Zingg et al., 2018) that appear to be largely conserved across species (see Buchanan and Johnson, 2011). Progress in understanding the complexities of the rodent claustrum have, however, been hindered by both its irregular shape as well as its small cross-sectional area, factors that have precluded, for instance, effective electrophysiological characterization. Progress has also been held back by a lack of clarity concerning the extent of its anatomical boundaries, an issue that is seated in the fact that rodents are lisencephalic and, as such, lack a well-defined extreme capsule (a structure that in gyrencephalic species provides a clear boundary between the claustrum and the neighboring cortex; for a recent review, see Smith et al., 2018). To overcome the problems that the resulting claustro-cortical continuity has presented, a sustained focus has been on identifying genes that show a differential expression profile in the claustrum relative to surrounding cortical areas. To this end, considerable progress has been made (Mathur et al., 2009; Mathur, 2014; Wang et al., 2017; Watakabe, 2017). Crystallin mu (Crym) expression, for instance, is densely expressed in the insular cortex yet is highly attenuated in the claustrum. Indeed, Crym expression was fundamental to establishing that the claustrum is surrounded on all sides by cortex rather than being juxtaposed with the external capsule (Mathur et al., 2009), as was thought previously. In the same study, the nuclear boundary of the claustrum at the level of the striatum was defined using the expression profiles of parvalbumin (PV), cytochrome oxidase, and the guanine nucleotide binding protein (G protein), gamma 2 (Gng2; Mathur et al., 2009). More recently, Wang et al. (2017) compiled a list of 49 genes that are differentially expressed in the mouse claustrum.

Alongside this progression, however, attempts to resolve the issue of whether, or not, the rostral boundary of the claustrum extends beyond the anterior aspect of the striatum have seen limited progress. In the seminal work of Mathur et al. (2009), the apparent absence of parvalbumin and Gng2 expression within the atlas-defined boundary of the rostral claustrum prompted a reassessment of the anatomical boundary of the claustrum to one that did not extend beyond the anterior horn of the neostriatum. Subsequent anatomical and behavioral studies have, for the most part, conformed to the Gng2-based anatomical definition of Mathur et al. (2009) (e.g., Smith and Alloway, 2010). In a recent review, however, Smith et al. (2018) highlight the importance of reaching a resolution in future studies. Indeed, in another recent review (Dillingham et al., 2017), using a freely available nucleotide sequence expression mouse brain database (Allen Mouse Brain Atlas^1^), the expression of a number of genes that were identified as having differential expression in the claustrum (Mathur et al., 2009; Wang et al., 2017) were assessed. Of 49 genes, the striatal – claustrum boundary, delineated either by attenuated expression (e.g., Slit-1, Crym), or enriched expression (e.g., Gng2, Gnb4, latexin), was found to extend considerably rostral to the striatum. Significantly, however, unlike atlas-based delineations, the oval cross section of the claustrum is situated at the ventrolateral aspect of the forceps minor of the corpus callosum, i.e., maintaining its locus in Layer 6 of the insular cortex. Given the multimodal nature of the claustrum (Remedios et al., 2010) and the likelihood that the separate “puddles” of (presumably functional) connectivity act in concert (Smythies et al., 2014), it is all the more important that a consensus in the field relating to its anatomical boundaries is reached.

In the present study, a combination of immunohistochemistry (including immunofluorescence) and pathway tracing was employed to examine the expression patterns of several genes that have been identified as verified claustral markers. One, crystallin mu (Crym), exhibits an attenuated expression in the claustrum relative to surrounding cortex (Mathur et al., 2009), while Gng2 (Mathur et al., 2009) and parvalbumin (Druga et al., 1993; Rahman and Baizer, 2007; Mathur et al., 2009; Hinova-Palova et al., 2014a; Pirone et al., 2015) show enriched expression in the claustrum. By reassessing the expression profiles of these genes while taking advantage of known, dense claustrum-cortical connectivity (through anterograde and retrograde pathway tracing) the focus of this study was on establishing the rostral boundary of the claustrum in the rat brain.

## Materials and Methods

### Subjects

A total of 43 male Lister Hooded rats (Envigo, United Kingdom) with pre-procedural weights of between 230–350 g were used in the study. In 6 animals, retrograde tracer injections targeted nucleus reuniens (RE) and/or the rhomboid (Rh) nucleus of the midline thalamus and in one of these cases, a further retrograde tracer injection targeted the mediodorsal (MD), centromedial (CM) and paraventricular thalamic nuclei (some of these cases used in Mathiasen et al., 2019; Table 1). In 2 of the animals with RE/Rh injections the injection site also included a portion of the centromedial thalamic nucleus. Further, in 18 animals, retrograde (*n* = 13) or anterograde (*n* = 7) tracer injections targeted the retrosplenial (RSC) or anterior cingulate (Cg) cortices (Table 1). In 6 of the animals with tracer injections we further immunoreacted for PV using immunofluorescence, while a further 16 animals were used only for immunohistochemistry (Table 1).

**Table 1:** An overview of the cases used in the study, including details of the type, and target of cortical and thalamic tracer injections.

Cases	Injection sites	Tracer	Immunofluo rescence
**Thalamic injections (retrograde tracers)**			
215#4	MD, PV, CM	CtB	
	RE/Rh	FB	
208#9	RE	FB	
207#2	RE/Rh (SMT, IAM)	FB	Parvalbumin
207#4	RE/Rh (CM, IMD)	FB	
207#7	RE (PVT, CM, IAM)	FB	
209#10	RE/posterior hypothalamus	CtB	
207#1	RE	FB	Parvalbumin
**Cortical injections (retrograde tracers)**			
199#29	RSC	FB	
225#4	RSC	CtB	
225#12	RSC	CtB	
223#1	RSC	CtB	
198#12	RSC	FB	
199#29	RSC	FB	
225#1	RSC	CtB	Parvalbumin
225#1	Cg	FB	Parvalbumin
222#10	RSC	CtB	
222#10	Cg	FB	
223#5	Cg	FB	
223#26	Cg	CtB	
FGrsc#1	RSC	FG	Parvalbumin
FGrsc#2	RSC	FG	Parvalbumin
LK#1	RSC	CtB	Parvalbumin
**Cortical injections (anterograde tracers)**			
224#30	RSC	AAV-CaMKIIa-EGFP, AAV5	
224#29	RSC	AAV-CaMKIIa-EGFP, AAV5	
219#2	Cg	AAV-CaMKIIa-EGFP, AAV5	
219#9	Cg	AAV-CaMKIIa-EGFP, AAV5	
219#3	Cg	AAV-CaMKIIa-hM4D(Gi)-mCherry, AAV5	Parvalbumin
219#17	Cg	AAV-CaMKIIa-hM4D(Gi)-mCherry, AAV5	
219#21	Cg	AAV-CaMKIIa-hM4D(Gi)-mCherry, AAV5	
**Immunohisto**	**Marker**	**Plane**	
**chemistry**			
HPCLA1	Crym, Gng2	Coronal	
HPCLA2	Crym, Gng2	Coronal	
RCLA1	Crym, Gng2	Coronal	
RCLA2	Gng2	Coronal	
RCLA3	Gng2, Crym	Coronal	
RCLA4	Gng2	Coronal	
RCLA5		Coronal	
SHPC1	PV	Coronal	
BiFlex1	PV, Crym	Coronal	
BiRCLA1	PV, Crym	Coronal	
BiRCLA2	PV	Coronal	
BiRCLA3	PV, Crym	Coronal	
CLArsc2	PV	Horizontal (axial)	
WK2	PV	Horizontal (axial)	
LH1	Gng2	Coronal	
LH2	Gng2	Coronal	


### Compliance With Ethical Standards

Animal husbandry and experimental procedures were carried out in accordance with the European Community directive, 86/609/EC, and the Cruelty to Animals Act, 1876, and were approved by the Comparative Medicine/Bioresources Ethics Committee, Trinity College, Dublin, Ireland, and followed LAST Ireland and international guidelines of good practice or, for those experiments that were performed at Cardiff University, in accordance with the United Kingdom Animals (Scientific Procedures) Act, 1986 and associated guidelines, the EU directive 2010/63/EU, as well as the Cardiff University Biological Standards Committee.

### Surgical Procedures

Anesthesia was induced and maintained with isoflurane (5% and 1–2%, respectively) combined with oxygen (2 L/min). Animals were then placed in a stereotaxic frame (Kopf, Tujunga, CA, United States) and chloramphenicol eye ointment (Martindale Pharmaceuticals, Romford, United Kingdom) was topically applied to the eyes to protect the cornea. Pre-surgical analgesia (Metacam, 1 mg/kg; Boehringer Ingelheim, Germany) and antibiotics (Enrocare; Animal Care Ltd., York, United Kingdom) were administered subcutaneously. The scalp was incised and cleaned before craniotomies were made, large enough to permit advancement of a Hamilton syringe into thalamic or neocortical regions. For retrograde tracing we injected either Fast Blue (FB; Polysciences), Fluorogold (FG; Santa Cruz Biotechnology, Heidelberg, Germany), or cholera-toxin b (CtB; List Biological Labs Ltd., California, United States). For anterograde tracing we injected an adeno-associated virus expressing either mCherry [AAV-CaMKIIa-hM4D(Gi)-mCherry, AAV5], or GFP (AAV-CaMKIIa-EGFP, AAV5) as a fluorescent marker (Addgene, Cambridge, MA, United States).

Following tracer-specific survival times, ranging from 5 days to 7 weeks (the latter for viral injections in anterior cingulate only), rats were deeply anesthetized with sodium pentobarbital (Euthanimal) and perfused transcardially with ice cold 0.1 M phosphate buffered saline (PBS) followed by either 2.5% or 4% paraformaldehyde in 0.1 M PBS.

### Immunohistochemistry (IHC)

In cases used for immunohistochemistry (with no tracer injections), the brains were removed and post-fixed for 48 h before being transferred to a 25% sucrose in 0.1 M PBS solution for 1–2 days for cryoprotection. Sections of 40 μm were cut (either in the coronal or horizontal plane; Table 1) on a cryostat (Leica CM1850) with one 1-in-4 series mounted directly on to double gelatine-subbed microscope slides. Of the remaining 3, 1-in-4 series, one or more of: an anti-Gng2 polyclonal antibody raised in rabbit (Sigma-Aldrich Ireland Ltd; Wicklow, Ireland), an anti-Crym monoclonal antibody raised in mouse (Novus Biologicals; Abingdon, United Kingdom), and an anti-parvalbumin monoclonal raised in mouse (Swant Inc., Marly, Switzerland) were used to immunolocalize the respective proteins.

Initially, endogenous peroxidases were removed from free-floating brain sections through reaction in a quench solution containing 10% methanol and 0.3% hydrogen peroxide in distilled water. Following washes in PBS and subsequently PBST (0.05% Triton X-100 in 0.1 M PBS), the sections were agitated in a 4% solution of normal horse serum in 0.1 M PBS for 2 h. Sections were then transferred to a 1:200 dilution of either anti-Crym or anti-Gng2 in 0.1 M PBST with 1% normal horse serum and agitated at 4^o^C overnight. Following washes in PBST, sections were transferred to a 1:250 dilution of biotinylated horse-anti-mouse IgG (for sections reacted against Crym; Vector Labs, Peterborough, United Kingdom) or biotinylated horse-anti-rabbit IgG (for sections reacted against Gng2; Vector Labs, United Kingdom) for 2 h. Sections were then washed in PBST before undergoing signal amplification through incubation in the Vectastain ABC solution (Vector Labs, Peterborough, United Kingdom) for 2 h. Following washes in PBST and subsequently PBS, sections were agitated overnight at 4^0^C. Immunoreactivity was visualized using the chromagen diamino benzidine (DAB; Vector Labs, Peterborough, United Kingdom) and in some cases, the signal was intensified with by adding nickel chloride to the DAB solution. Sections were then washed in PBS, mounted, and left to dry at room temperature before being dehydrated in ascending alcohols, cleared in xylene, and coverslipped with DPX mounting medium (Sigma-Aldrich, Gillingham, United Kingdom).

### Immunofluorescence Staining

In cases with anterograde or retrograde tracer injections, brains were post-fixed for 4 h before being transferred to a 25% sucrose solution overnight. Sections of either 40 μm or 50 μm were cut in the coronal plane with a freezing microtome with one 1:4 series used for Nissl staining, a second series used for visualization of the tracers and, in some cases, remaining series were used for further immunofluorescence staining (Table 1).

For visualization of tracers, brain sections were washed in PBS and PBST followed by incubation with the relevant primary antibody (rabbit anti cholera toxin (1:3000-1:10000 dilution; Sigma-Aldrich United Kingdom) or rabbit anti-mCherry (1:2000 dilution; Abcam, Cambridge, United Kingdom) overnight. Following PBST washes the sections were incubated with the secondary antibody (1:200 dilution; goat anti-rabbit DyLight 594; Vector Laboratories; Peterborough, United Kingdom), washed in PBS, mounted and coverslipped either directly with Fluoromount (Sigma-Aldrich, Gillingham, United Kingdom) or alternatively, following dehydration in ascending alcohols, with DPX mounting medium (Thermo Fisher Scientific, Waltham, MA).

In a number of these cases (see Table 1) sections were further stained for mouse anti-parvalbumin, (1:10000 dilution; Sigma-Aldrich, United Kingdom) in a 1% NGS (Vector Labs, Peterborough, United Kingdom) PBST solution following 90 min in a 5% NGS solution. Sections were incubated overnight, washed in PBST and incubated with the relevant secondary antibody (goat anti-mouse Alexa Fluor 488/594 (Abcam, Cambridge, United Kingdom) in 1% NGS 1:200 PBST.

## Microscopy and Imaging

Brain sections were imaged in brightfield at 20× magnification using a Leica slide scanner (Aperio AT2), visualized in Aperio ImageScope (version 12.3.2.8013). For fluorescence microscopy two systems were used. Either a Leica DM6000 B microscope with an attached Leica DFC350 FX digital camera with acquisition software (LAS AF image, Leica), or a Leica DM5000 B microscope with an attached Leica DFC310 FX digital camera. Images were adjusted for brightness and contrast in Corel Photo Paint X5 or FIJI (“*fiji is just imageJ*” freely available software^2^). Pixel density heat maps were generated in Fiji; images were converted to 8-bit and median filtered before applying a 16-color LUT.

### Nomenclature

Based on their recent guidance and clarification on the issue of how to consider the claustrum in relation to the endopiriform nuclei, we follow the classification of Smith et al. (2018) and consider a claustrum-endopiriform complex comprising the claustrum proper and the dorsal endopiriform nucleus (DEn), with the claustrum proper comprising dorsal (dCLA) and ventral (vCLA) subdivisions. Delineations of the vCLA/dCLA border was estimated based on comparison of gene markers that are specific to vCLA (PV) with those that show differential expression in both vCLA and dCLA (Crym and Gng2). In addition to atlas-based (Paxinos and Watson, 2005) definitions of the insular/orbital region, Nissl stained sections were used to delineate the border between the insular and orbital cortices. The lateral orbital cortex displays prominent cytoarchitectonic differences from the insular cortex, such as a more densely packed layer 5 and a less sharp border between layers 2 and 3 (Van De Werd and Uylings, 2008).

## Results

### Anatomical Boundary – Parvalbumin (PV) (IHC)

#### Coronal Plane

**FIGURE 1:**
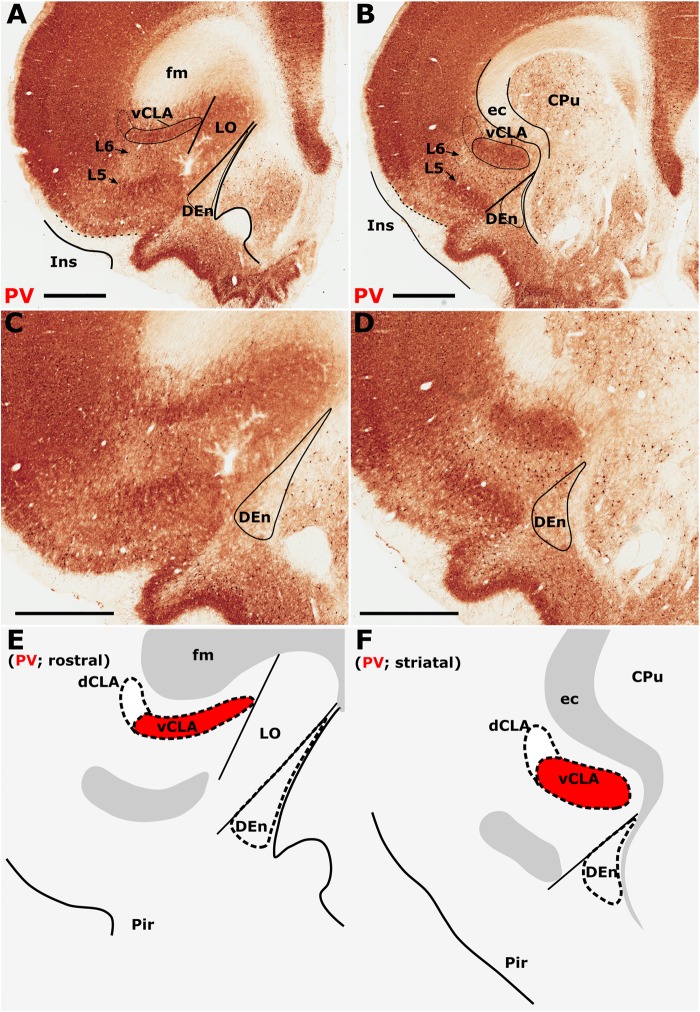
Differential parvalbumin (PV) expression in the insular region aides the delineation of the anatomical border of the claustrum. **(A–D)** show low **(A,B)** and high **(C,D)** magnification photomicrographs of PV immunoreactivity in brain sections rostral to the striatum **(A,C)** and at the level of the striatum **(B,D)**. At the level of the striatum, dense expression of PV neuropil in the ventral claustrum (vCLA) contrasts with weaker expression in the neighboring layer 6 of the insular cortex **(B,D)**. Rostral to the striatum, dense PV neuropil is again observed deep to layer 6 of the insular cortex (Ins). PV is expressed in the vCLA but does not allow for the delineation of the borders of the dorsal endopiriform nucleus (DEn), or the dorsal claustrum (dCLA) whose boundaries are estimated in **(C,D)**. Images **(E,F)** are schematic representations of **(C,D)**, respectively. Red fill represents the part of the complex that can be delineated using PV immunolocalization. Note in **A** and **C** that due to dense expression in the lateral orbital cortex (LO), the border between the medial extent of vCLA and LO is not easily determinable (asterisk). CPu, caudate/putamen; ec, external capsule; fm, forceps minor of the corpus callosum; Pir, piriform cortex. Scale bars = 1000 μm.

Parvalbumin expression in the neuropil of the agranular insular agranular insular cortex is characterized by attenuated expression with the exception of a densely labeled fiber plexus in layer 5 (Figure 1A–D). Contrasting dense expression of PV was present in the neuropil of the vCLA (Smith et al., 2018). PV-immunoreactive neuron density was found to be sporadic but uniformly distributed across the insular cortex and vCLA with no discernible inter-laminar difference (Figure 1C,D).

**FIGURE 2:**
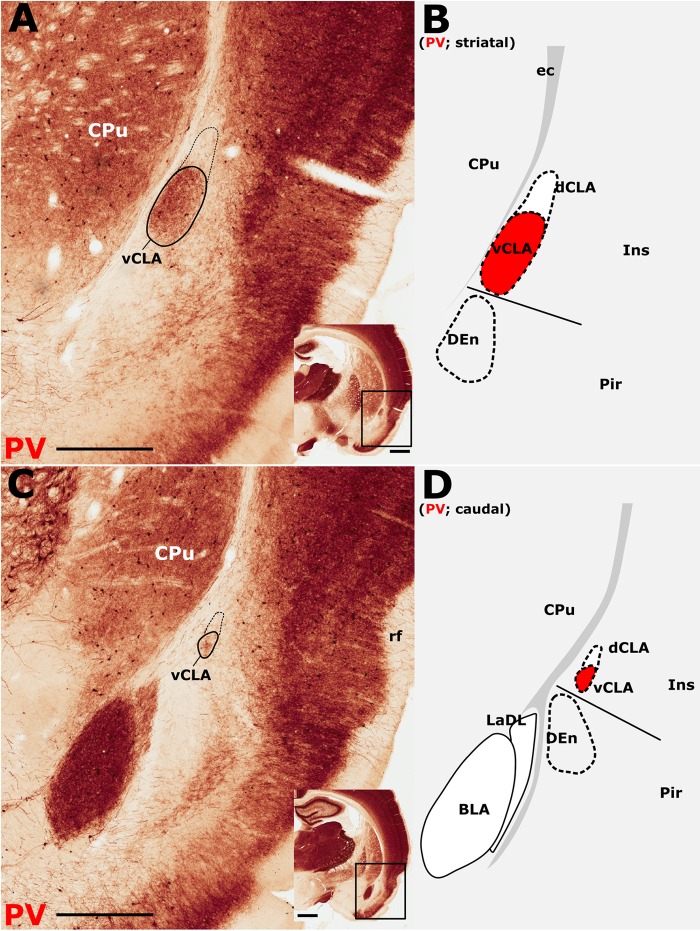
Parvalbumin (PV) expression showing the caudal extent of the ventral claustrum (vCLA). **(A)**, At a level approximately –1.8 mm from bregma (see low magnification inset), the claustrum is situated within layer 6 of the insular cortex (Ins). Note the absence of label in the dorsal claustrum (dCLA). **(B)**, Further caudally, at the level to the anterior amygdala complex (approximately –2.5 mm from bregma; see low magnification inset), the claustrum is significantly reduced in cross sectional area. The vCLA was not present caudal to this coronal section. Schematic representations of **A** and **C** are shown in **B** and **D**, respectively, with red fills representing PV expression in the claustrum. BLA, basolateral amygdala nucleus; CPu, caudate putamen; DEn, dorsal endopiriform nucleus; ec, external capsule; Ins, insular cortex; LaDL, lateral amygdaloid nucleus, dorsolateral part; Pir, piriform cortex. Scale bars in **A,C** = 500 μm; insets = 1000 μm.

The insular cortex is bordered caudally by the peri- and ectorhinal cortices, while the rostral boundary of the insular cortex interfaces with the orbital cortices (Figure 1A,C). Both orbital and rhinal cortices regions exhibit a uniformly higher density of PV immunoreactivity across the neuropil of layers 4–6, albeit again with increased expression in layer 5. The transition of insular to peri/ectorhinal cortex matches closely with the caudal apex of the vCLA (Figure 2), i.e., a continuous extension of claustral PV expression into the rhinal cortices was not present.

At the anterior horn of the neostriatum, PV expression in vCLA remained dense with no apparent reduction in cross-sectional area. At this coronal level (approximately represented by the +2.52 (from Bregma) plate in Paxinos and Watson, 2005), the ovoid cross-section of the claustrum is more horizontally oriented (Figure 1), and elongated within the arch of the external capsule. Immediately rostral to the anterior horn of the neostriatum, the putative vCLA was still present, maintaining its position beneath the forceps minor of the corpus callosum (within layer 6 of the insular cortex). Further rostrally, the lateral orbital cortex emerges to laterally displace both the insular cortex and the vCLA to a position progressively more lateral with respect to the forceps minor (Figure 1A,C).

In our analyses of PV across multiple coronal cases, the vCLA was consistently found to extend approximately 500 μm anterior to the anterior horn of the striatum. It is worthy of note that PV expression in the lateral orbital cortex was uniformly dense across its layers, such that the medial extent of the vCLA and the lateral orbital cortex appeared continuous; it was therefore difficult to determine this border between regions (Figure 1A,C).

#### Horizontal (Axial) Plane

**FIGURE 3:**
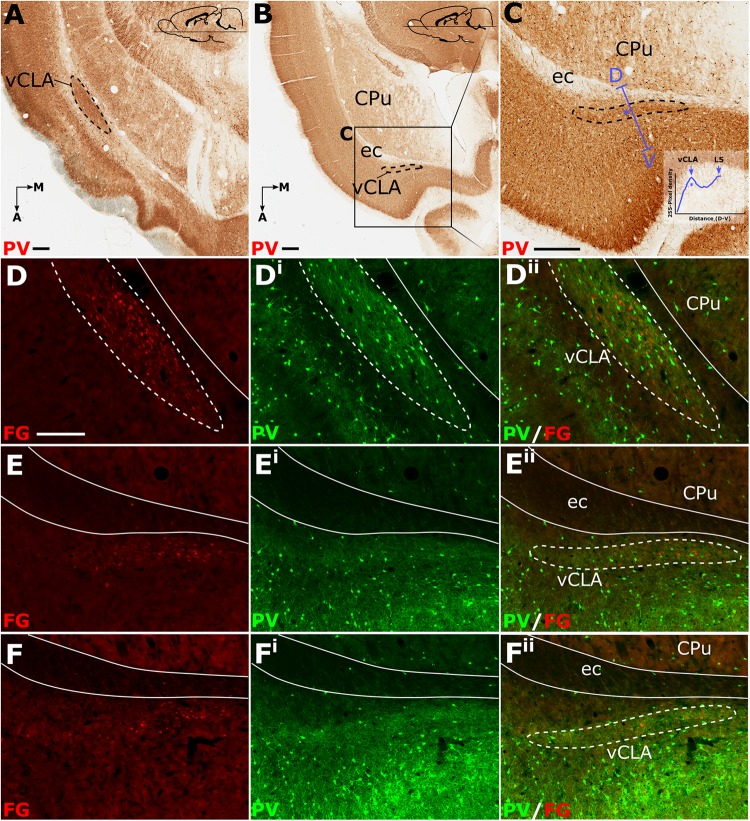
Parvalbumin (PV; pseudocolored green) expression in horizontal sections **(A–C)** provided complementary findings to those derived from coronal sections. Unlike coronal or sagittal series, the horizontal plane allows for visualization of much of the anterior-posterior extent of the ventral claustrum (vCLA) in a single section **(A)**. Low magnification **(B)** and high magnification **(C)** images of the rostral extent of the vCLA (dashed line) in a comparatively more dorsal section than **A**. Dual fluorescence, combining PV expression (green) and retrograde labeling following injections of Fluoro-gold into the retrosplenial cortex (pseudocolored red), showed that, at striatal levels **(D–D^ii^)**, corresponding to that in **(A)**, dense retrograde label overlaid claustral PV enrichment. Rostral to the striatum **(E–E^ii^, F–F^ii^)** retrograde labeled cell soma again overlaid parvalbumin enrichment corresponding to the claustrum, albeit with fewer retrogradely labeled soma and weaker PV expression. Scale bar in **A–C** = 500 μm. Scale bar in **D** (applies to all fluorescent images) = 250 μm.

Relative to the midline, the position of the claustrum courses approximately 2 mm medially from its caudal position at bregma to its rostral position at the anterior horn of the neostriatum (Paxinos and Watson, 2005), such that visualization of the nucleus in the true-sagittal plane is only moderately beneficial in examining its continuity in the rostro-caudal axis. The dorsal-ventral position of claustrum, however, remains relatively consistent along this rostro-caudal extent such that visualization of large portions of its continuity in the same plane of section is possible (e.g., 2 mm in Figure 3A). Retrograde tracer injections (Fluoro-gold) into the retrosplenial cortex were combined with PV immunofluorescence in horizontal brain sections to further assess the rostral extent of vCLA. At striatal levels both PV expression and distributions of retrograde cell soma label clearly demarcated the claustral area. Beyond the anterior horn of the neostriatum, the vCLA arches upward beneath the forceps minor. As a result, retrograde label and PV expression were observed in comparatively dorsal horizontal sections (Figure 3B,C). At these dorsal levels, unlike in coronal sections in which contrast is present between dense claustral PV expression and weak expression in the immediately adjacent layer 6 of the insular cortex, the claustrum in horizontal sections is bordered by more comparably dense cortical PV expression. At both striatal levels (Figure 3D–D^ii^), and rostral to the striatum (Figure 3E–E^ii^,F–F^ii^), retrogradely labeled cell bodies were present in the claustrum and in distributions that closely matched claustral PV expression.

### Anatomical Boundary – Crystallin mu (Crym) (IHC)

**FIGURE 4:**
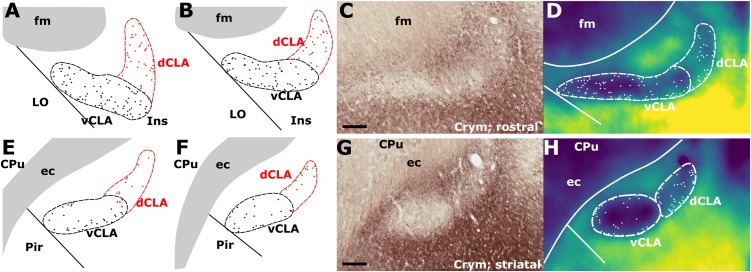
**(A,B)** Schematic representations of crystallin mu (Crym) delineated boundaries of the claustrum anterior to the striatum (CPu), highlighting the density of ectopic Crym-positive cell soma within the ventral (vCLA; black) and dorsal claustrum (dCLA; red). **(C)** shows representative Crym staining in the claustrum/insular while **(D)** shows a pixel density heat map of **(C)** highlighting 1. The difficulty associated with determining the boundary between vCLA and dCLA; and 2. Cortical fibers crossing the claustrum to join the internal capsule. **(E–H)** show equivalent panels from striatal levels in which the number of ectopic Crym-positive cell soma is reduced and the boundary between vCLA and dCLA is more distinct. ec, external capsule; fm, forceps minor of the corpus callosum. Scale bars = 200 μm.

**FIGURE 5:**
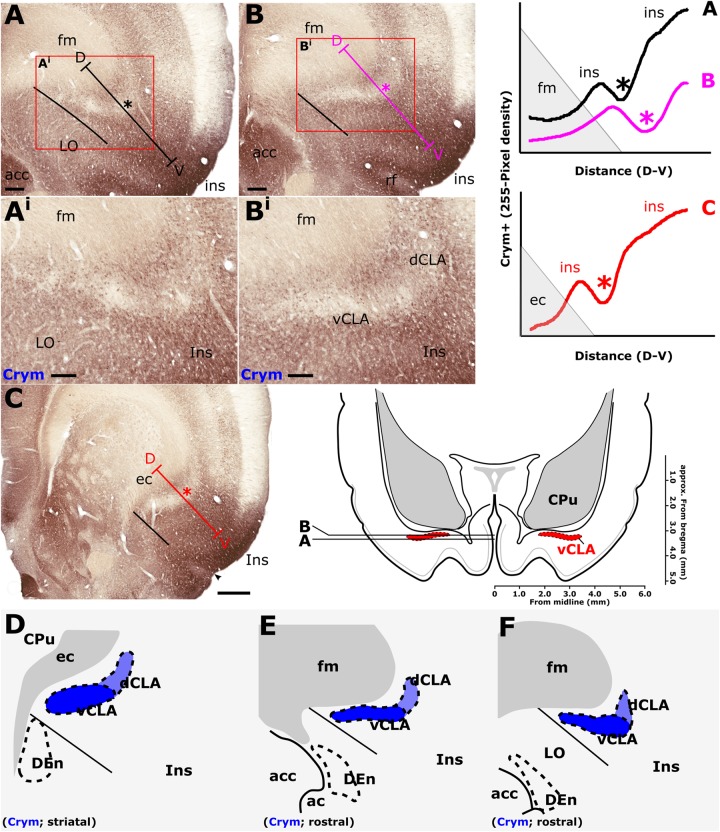
Crystallin mu (Crym) is a cortical marker that is expressed particularly strongly in the insular cortex. In contrast, expression in the claustrum is considerably reduced providing contrast for delineation of the anatomical boundary of the claustrum. Pixel density plots through the insular cortex at both striatal levels (**C**; red), and rostral to the striatum (**A,B**; black and blue, respectively) show cortical peaks either side of a claustral trough (asterisks). Central schematic diagram shows approximate coronal levels of photomicrographs in **(A,B)**. **(D–F)**: Schematic representations of Crym-based delineation of the claustrum-endopiriform complex. Delineation of the dorsal endopiriform nucleus (DEn) is not possible using Crym, however, vCLA, and to a lesser degree dCLA are (See also Figure 3). acc, nucleus accumbens; CPu, caudate/putamen; ec, external capsule; fm, forceps minor of the corpus callosum; Ins, insular cortex; LO, lateral orbital cortex. Scale bars in **A,B** = 300 μm; **A^i^,B^i^** = 200 μm; **C** = 600 μm.

In findings that are consistent with reports in the mouse (Wang et al., 2017) and rat (Mathur et al., 2009), expression of Crym was dense in the insular cortex at striatal levels of the telencephalon but markedly reduced in the vCLA (Figure 4C,G, 5). In the putative dCLA, Crym-immunoreactive neuropil was reduced but to a lesser degree, while DEn was not discernible as the intensity of Crym immunoreactivity was similar to that in the neighboring piriform cortex (Figure 5A–C). Within the insular region, particularly high densities of Crym-immunoreactive cell bodies and neuropil were distributed around the circumference of the vCLA/dCLA complex. Within the vCLA and dCLA, the distribution density of Crym-immunoreactive cell bodies was considerably reduced with just a few scattered ectopic Crym-positive soma (Figure 4E–H, 5), although rostral to the striatum, the density of these “ectopic” cortical soma was higher (Figure 4A–D).

Consistent with past dual-immunofluorescence (Crym and PV) experiments (Mathur et al., 2009), differential expression of Crym in vCLA relative to the surrounding insular cortex delineated an anatomical boundary that closely matched that derived from our PV expression profile (Figure 5C–E and Supplementary Figure S1), forming an increasingly elongated ovoid cross-section in the coronal plane toward the anterior horn of the neostriatum. Beyond the striatum, the Crym-based vCLA boundary formed a horizontally oriented ovoid beneath the forceps minor of the corpus callosum while further rostrally it was found to apex ventrolaterally beneath the forceps minor (while remaining confined to the boundary of the insular cortex; Figure 5D–E). Unlike the PV expression profile, however, the Crym profile enabled a clear delineation of the boundary between the vCLA (weak Crym expression) and the lateral orbital cortex (dense Crym expression), with the finding that vCLA did not extend into the lateral orbital cortex but remained confined to the boundaries of the insular region (Figure 5A,B). Crym expression was also found to be reduced in dCLA (Figure 5), which meant that the precise vCLA-dCLA transitional boundary was not clear; an issue that was also contributed to by the presence of Crym-immunoreactive fibers ascending to the internal capsule (Coizet et al., 2017; Figure 5A^i^,B^i^).

### Anatomical Boundary – Gng2 (IHC)

**FIGURE 6:**
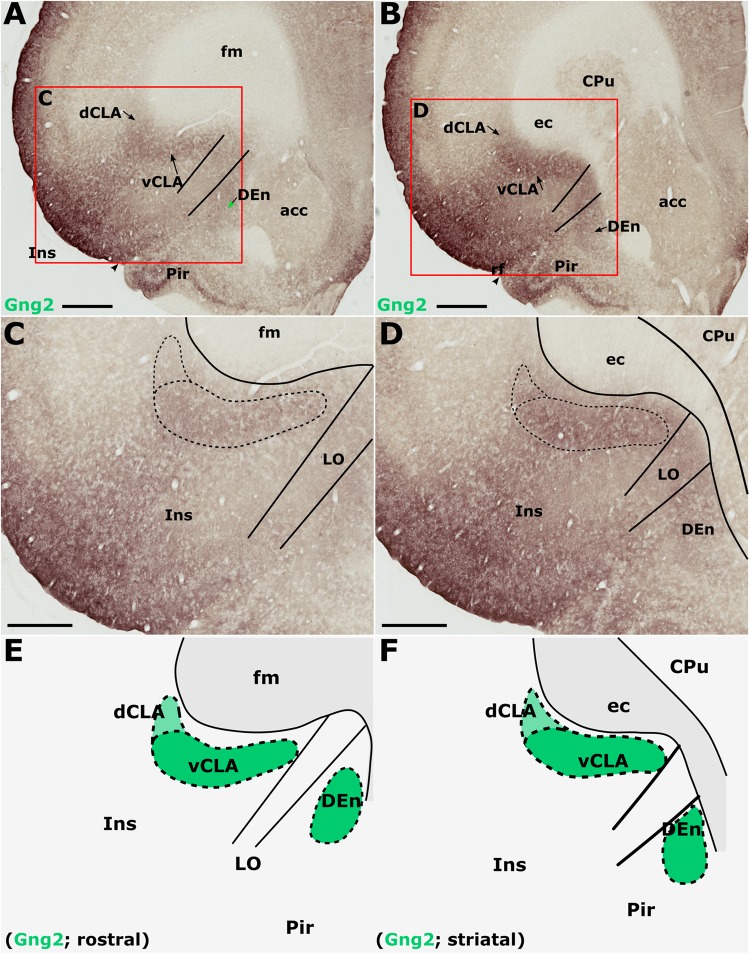
Guanine nucleotide binding protein (G protein), gamma 2 (Gng2), is expressed throughout the insular cortex but relatively weakly in layers 5/6. At striatal levels **(B,D)**, dense expression is observed in the ventral claustrum (vCLA) and dorsal endopiriform nucleus (DEn). Expression in the dorsal claustrum (dCLA) but it is relatively weaker **(D)**. The same distribution of expression is evident anterior to the striatum **(A,C)** albeit with weaker expression throughout the claustrum-endopiriform complex. Note in **A** and **C**, the separation of DEn from vCLA with the emergence of the lateral orbital cortex (LO). **(E,F)** are schematic representations of Gng2 expression (green) rostral to the striatum and at striatal levels, respectively. acc, nucleus accumbens; CPu, caudate/putamen; ec, external capsule; fm, forceps minor of the corpus callosum; Ins, insular cortex; Pir, piriform cortex. Scale bars in **A,B** = 800 μm; **C,D** = 500 μm.

Gamma 2 immunoreactivity was found to be densely distributed throughout the neuropil of the insular cortex (Figure 6A–D). The densest Gng2-immunoreactivity was present in the superficial-most layers and was reduced in layers 5 and 6 which contrasted with the dense vCLA immunoreactivity (Figure 6A–D). Dense expression was observed in layer 2 of the piriform cortex but weak expression in layer 3 again provided contrast with denser Gng2 immunoreactivity in DEn (Figure 6A–D).

Gamma 2 immunoreactivity delineated a vCLA boundary that was consistent with both PV and Crym, albeit with a less-well pronounced margin (Figure 6E,F). Indeed, manual registration of serial sections that had been immunohistochemically (DAB) reacted for either Crym or Gng2, revealed expression profiles of vCLA and dCLA Gng2 enrichment that closely matched (at all claustral levels) the region of Crym attenuation in the corresponding section (see Supplementary Figure S2). At striatal levels, Gng2 enrichment in DEn was continuous ventrally with vCLA although with denser expression in vCLA (Figure 6B,D), so that the boundary between the two nuclei at the piriform/insular boundary was distinct. Rostral to the anterior horn of the striatum, DEn was no longer continuous with vCLA, and the two regions became progressively separated by the emergence of the lateral orbital cortices, i.e., the extent of vCLA and DEn remained confined to insular and piriform cortices, respectively (Figure 6A,C,E). At this anterior-posterior level, Gng2 expression in vCLA formed a horizontally-oriented ovoid beneath the forceps minor of the corpus callosum with the dCLA arching around its ventrolateral border. As with PV and Crym, further rostrally, vCLA became more restricted in cross-sectional area and situated more laterally with respect to the forceps minor.

### Anatomical Boundary – Tracer Injections

Pressure injections of either retrograde (FB, CtB, or FG) or anterograde (viral) neuronal tracers were made targeting either the retrosplenial or anterior cingulate cortices, revealing a consistently dense pattern of label along the rostro-caudal extent of vCLA (see Table 1).

**FIGURE 7:**
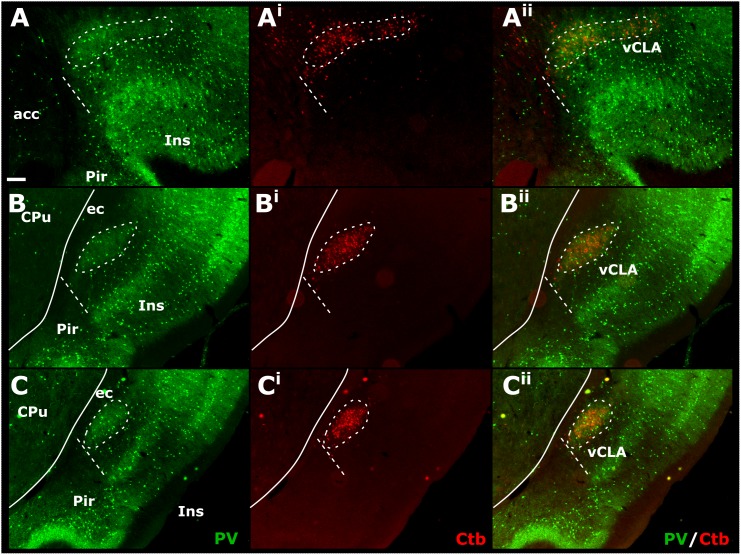
Tracer injections of the non-toxin subunit B of cholera toxin (CtB) within the retrosplenial cortex resulted in dense cell soma label in the ipsilateral claustrum in a distribution that overlapped with claustral (vCLA) parvalbumin (PV) expression (for a comparable case using Fluoro-gold, see Supplementary Figure S4 and for injections sites see Supplementary Figure S5). At anterior-posterior (AP) levels rostral to the anterior horn of the neostriatum (CPu, **A–A^ii^)** parvalbumin and CtB label delineated a claustral border (white dashed line) that was horizontally oriented within the arch of the forceps minor of the corpus callosum (fm). At rostral and mid-striatal AP levels (**B–B^ii^**,**C–C^ii^**, respectively), the claustral border was more vertically oriented alongside the external capsule (ec). acc, nucleus accumbens; CPu, caudate/putamen; ec, external capsule; Ins, insular cortex; Pir, piriform cortex. Scale bar = 200 μm.

Cases in which multiple FB, FG, or CtB injections were made unilaterally along the extent of the retrosplenial cortex or anterior cingulate cortex (more confined injections) resulted in dense retrograde label in the ipsilateral claustrum. Although weak, retrograde label was present in the claustrum of the contralateral hemisphere at both striatal levels, as well as rostral to the striatum (see Supplementary Figure S3). The distribution of retrogradely labeled cell bodies was confined to the ventral claustrum, i.e., it did not extend into the dorsal claustrum, or ventrally to the DEn (see below). Significantly, the distribution of retrograde label in vCLA extended beyond the anterior horn of the neostriatum, delineating a boundary consistent with that determined from IHC analyses (Figure 7A–A^ii^). In cases involving retrograde injections targeting the anterior cingulate cortex, dense cell labeling was present in the claustrum between 0.4 and 0.6 mm rostral to the anterior tip of the striatum. The cell labeling that resulted from injections in the retrosplenial cortex extended to comparable rostral levels, although with varying cell density.

**FIGURE 8:**
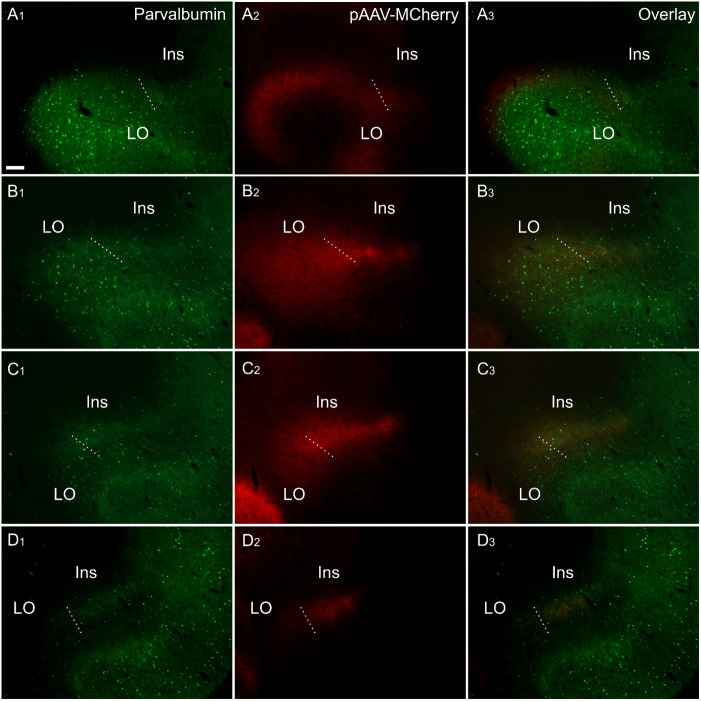
Fiber label resulting from multiple injections (bilateral) of an anterograde viral tracer [AAV-CaMKIIa-hM4D(Gi)-mCherry] in the anterior cingulate cortex (case 219#3; column 2) shown in sections co-labeled for parvalbumin (column 1). The four rows **(A–D)** show photomicrographs of the claustrum at four anteroposterior levels separated by 200 μm. Row **(D)** is at the most rostral portion of striatum, Rows **(C–A)** rostral to striatum. Terminal fiber label co-localized with parvalbumin (PV) in a plexus that extends at least two sections rostral to striatum. The dotted line indicates the border between lateral orbital and insular cortices. Ins, insular cortex; LO, lateral orbital cortex. “AAV-mCherry” is an abbreviation for AAV-CaMKIIa-hM4D(Gi)-mCherry. Scale bars = 200 μm.

Injections of anterogradely transported AAV-CaMKIIa-EGFP or AAV-CaMKIIa-hM4D(Gi)-mCherry (both serotype 5) confined to the anterior cingulate cortex (bilaterally in all cases; see Table 1), resulted in dense fiber/terminal labeling along the rostro-caudal extent of the claustrum (Figure 8), revealing a rostral extension of the claustrum beyond the anterior horn of the neostriatum that closely matched the distribution of retrograde label observed in CtB and FB cases (no differences were observed in the distribution of fiber labeling for the two different viral tracers). In these cases, however, the dense “plexus” of fiber label in the claustrum (deep to the insular cortex) was continuous with more widespread, diffuse fiber labeling in the orbitofrontal cortex which, rostral to the level of claustrum, centered in a fiber plexus in the deepest lamina of the lateral orbital cortex together (including more superficial labeling; Figure 8A_1_–A_3_,B_1_–B_3_). This orbital portion correspond to what has previously been suggested to constitute the rostral portion of the claustrum (Paxinos and Watson, 2005). In two further cases, injection of AAV-CaMKIIa-EGFP (serotype 5) into the retrosplenial cortex (unilaterally) resulted in a more restricted fiber distribution, as no dense fiber label was present in the lateral orbital cortex.

In representative cases in which either retrograde (CtB, FG or FB), or anterograde [AAV-CaMKIIa-hM4D(Gi)-mCherry] injections were made into the retrosplenial cortex (CtB) or anterior cingulate cortex (viral tracer and FB), we reacted the sections for PV (see Figure 8 for the anterograde tracing). In these dual- fluorescence cases, immunolocalization distributions of tracer label again closely matched PV immunoreactivity in the neuropil of the vCLA. The distribution of retrograde labeled cell bodies in CtB and FB cases, as well as anterograde fiber/terminal label in DREADDs-mCherry cases, rostral to the anterior horn of the neostriatum, was closely aligned with our PV-based definition of the rostral claustral area, as described above. Interestingly, the fiber label in the deep layer 6 of the lateral orbital cortex, which resulted from anterograde tracer injections in the anterior cingulate (see above), was shown to a large extent to overlap with a portion *devoid* of PV neuropillar label (Figure 8A_1-3_).

### Anatomical Boundary – Thalamocortical Connectivity

**FIGURE 9:**
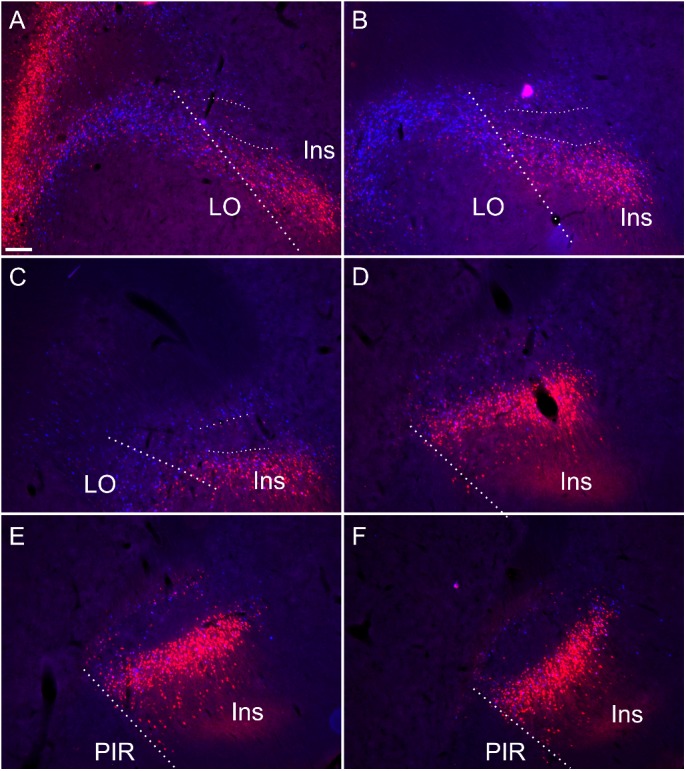
Retrograde soma label, in the insular and orbital cortices, resulting from cholera-toxin b (CtB, red) and Fast Blue (FB, blue) injections centered in mediodorsal/paraventricular/centromedial thalamic nuclei and nucleus reuniens/rhomboid (see Supplementary Figure S5 for injection sites corresponding to this case). Rostral to the striatum **(A,B)** and at anterior through to mid-striatal anterior posterior levels **(C–F)** the dense soma label encapsulates the claustral area where cell label is substantially attenuated **(A–F)**. Thick dashed lines demarcate the border between the orbitofrontal and insular cortices, while the thin dashed lines designate the approximate borders of the claustrum at rostral levels. Ins, insular cortex; LO, lateral orbital cortex; PIR, piriform cortex. Scale bars = 200 μm.

Retrograde tracer injections (FB or CtB; see Table 1), centered in the nucleus reuniens/rhomboid nuclei of the midline thalamus, resulted in dense retrograde label in the insular cortex. A comparable pattern of labeling was seen following an injection centered in the mediodorsal, paraventricular and centromedial thalamic nuclei (Figure 9; see also Supplementary Figure S5).

At striatal levels, a band of retrogradely labeled cell bodies was present in the insular cortex surrounding the claustrum, both superficial, i.e., juxtaposed to the external capsule and deep to the claustrum. Within the claustrum, very few retrogradely labeled cell bodies were present, particularly at more septal/striatal levels. Anterior to the striatum, the distribution of cortical label outlined a region of attenuated label that closely matched that which was defined by the differential expression of cortical tracers, Gng2, PV and Crym (Figure 9). In two of these cases, stained sections for PV confirmed that the region of attenuated label was indeed claustrum. In these same two cases overlays with Cresyl Violet-stained section confirmed that the border between the lateral orbital and the insular cortices co-localize with the PV based definition of claustrum.

## Discussion

A consensus on the anatomical boundary of the claustrum-endopiriform complex is important for establishing its functional role and, on a more immediate and practical level, for both the interpretation of, e.g., anatomical studies, as well as in the verification of electrode placements in electrophysiological studies.

**FIGURE 10:**
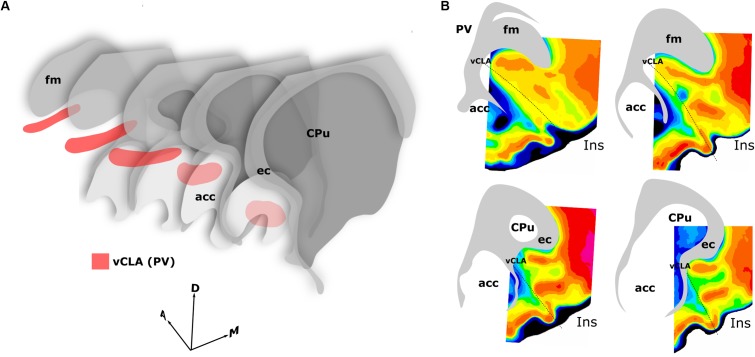
Schematics summarizing the rostral extent of the anatomical boundary of the claustrum in the rat. Expression of parvalbumin, crystallin mu, and Gng2, along with anterograde and retrograde tracing of claustro-cortical connectivity, and retrograde tracing of corticothalamic connectivity provided highly complementary definitions of the boundary outlined in **(A,B)**. **(A)** 3-dimensional representation of parvalbumin expression (red) in the ventral claustrum from caudal striatal (CPu) anterior-posterior levels (front) to rostral levels anterior to the striatum (back). Note the change in orientation of the long axis of the claustrum as it follows the arch of the forceps minor of the corpus callosum (fm) and the external capsule (ec). **(B)** Pixel density-based heatmaps (warm colors represent high expression) of parvalbumin expression in sections anterior to the striatum (upper) and at the level of the striatum (lower), reinforcing the extent and continuity of the claustrum rostral to the striatum. acc, nucleus accumbens; Ins, insular cortex.

Our primary finding is that the expression profiles of three claustral marker genes, Gng2 (Figure 6), PV (Figure 1) and Crym (Figure 5), as well as cortical (Figure 7, 8) and thalamic (Figure 9) tracing data, demonstrate that the anatomical boundary of the rat claustrum extends approximately 500 μm rostral to the anterior horn of the neostriatum, remaining confined throughout its rostro-caudal span to layer 6 of the insular cortex (Figure 10). Our findings relating to the *caudal* extent of the claustrum in the rat are in close accordance with atlas-based delineations (e.g., Paxinos and Watson, 2005), where vCLA and dCLA terminate at the level of the transition of insular to rhinal cortices (Figure 2). Caudal to this coronal level, the claustral differential expression profiles of Crym, Gng2, and PV within the deep cortex were no longer apparent.

In findings that are consistent with reports both in the mouse (Wang et al., 2017) and rat (Mathur et al., 2009), expression of Crym was dense in the insular cortex. At striatal levels, dense distributions of Crym-immunoreactive cell bodies and neuropil were distributed around the circumference of the claustrum, very clearly delineating the cortical shell surrounding the claustrum as described previously (Mathur et al., 2009). Within the boundary of the vCLA, Crym-immunoreactivity (both cell bodies and neuropil), was all but absent except for a handful of Crym-immunoreactive (putative) ectopic cortical cell bodies. Conversely, Gng2 and PV expression was enriched within the vCLA and delineated a boundary that closely matched that which was negatively outlined by the Crym expression profile. Mathur et al. (2009) established, through dual-immunofluorescence, that PV expression in the claustrum revealed an anatomical boundary of the claustrum that closely matched that shown by Crym (Figure 10). Using manual registration of serial sections, we have shown that Crym and Gng2 outline a similarly consistent nuclear boundary, providing further validation of these markers (Supplementary Figure S2). Retrograde cortical and thalamic tracing experiments, as well as anterograde cortical tracing cases provided data that was highly complementary to our IHC findings. The rostral claustrum has been suggested to be positioned deep to the ventral and lateral orbital cortices (Paxinos and Watson, 2005). We observed that fibers from the anterior cingulate cortex terminated densely in this area but, importantly, that this fiber plexus did not co-localize with the PV label, thereby further consolidating the idea that this area is cortical and not claustral.

Numerous studies have reported dense connectivity between the claustrum and the thalamus in the rat (Herkenham, 1978; Zhang et al., 2001; McKenna and Vertes, 2004; Yoshida et al., 2005; Vertes et al., 2006, 2012). Given the presence of ectopic cortical neurons throughout the rostral-caudal extent of the claustrum (Mathur et al., 2009), there is uncertainty as to whether these reported connections are accurate. Our data seems to support the case presented initially by Mathur et al. (2009) that the cortex immediately surrounding the claustrum shares subcortical connectivity but the claustrum itself does not. Our injections of retrograde tracer into the thalamus included the three thalamic nuclei that have been suggested to receive claustral afferents (Herkenham, 1978; Zhang et al., 2001; Erickson et al., 2004; McKenna and Vertes, 2004; Vertes et al., 2012). We did observe scattered thalamic projecting cells in the claustrum area, especially at rostral level. However, as the same pattern was seen in Crym stained sections, i.e., ectopic insular cortical neurons were occasionally scattered within the claustrum, with the same rostro-caudal distribution, it is most likely that our retrogradely labeled cells were in fact cortical cells.

In certain respects, our findings contradict those of Mathur et al. (2009), whose conclusions prompted a reassessment of the atlas-based anatomical boundary of the claustrum to one which: 1. Did not extend rostral to the anterior horn of the neostriatum, and 2. Was not juxtaposed to the external capsule, but was instead surrounded by a cortical shell.

In their study, Mathur et al. (2009) also examined the expression of PV, Gng2, and Crym in the rat, using the same primary antibodies and similar dilutions. However, as most data were shown as immunofluorescence label, it is possible that our avidin-biotin-based peroxidase approach was more sensitive to identifying the slightly weaker frontal signal (in the Gng2 stain). Additionally, in their analysis of Gng2 and PV expression, photomicrographs depict an absence of label in the region ventral to the forceps minor of the corpus callosum, but one that is at an extreme rostral level in which this region is orbital, not insular (Mathur et al., 2009). The level depicted represents the rostral-most extent of the insular cortex at which level it is situated more laterally, i.e., outside of the presented field of view. In the same study, tract tracing was used in combination with PV immunofluorescent localization and, in this instance, images were centered over PV immunofluorescence in the orbital cortices, in which no retrograde label was observed. It would, therefore, seem to be the case that Mathur et al. (2009) were correct in their disagreement with the atlas of Paxinos and Watson (2005), in that the claustrum is not situated within the orbital cortex at rostral levels, but mistaken in their conclusion that the claustrum was, therefore, only present at striatal levels. The consequence of these contradictory findings has been the development of a trend in many recent studies to include a methodological note stating that analyses *of claustral* labeling *did not extend beyond the most rostral coronal section that contained striatum due to the reported absence of Gng2 expression in these regions* (e.g., Smith and Alloway, 2010), contributing to an incomplete understanding of the claustrum.

As mentioned, the differential expression of Gng2 and Crym in the frontal extension of the claustrum becomes less accentuated. It would seem to be the case that toward the rostral apex of the claustrum, the density of ectopic cortical neurons within the claustrum increases, constituting something of a claustro-cortical transition (Figure 4 and Supplementary Figure S1), but it is also worthy of note that at these rostral levels, ascending axon bundles from neurons within the insular/orbital cortices enter the forceps minor of the corpus callosum in a path that bisects the claustrum (Coizet et al., 2017). These bundles would appear to reduce both the uniformity of Crym attenuation and the clarity of the gene marker-defined boundary.

Parvalbumin expression in the rodent CLA-DEn complex is confined to the vCLA (Smith et al., 2018), avoiding the DEn and the dCLA. As a result, the distribution of PV provides an important reference in determining the extent of gng2 and Crym expression within the CLA-EN complex and, of relevance here, the relative components of the rostral extent of the complex. At the anterior horn of the neostriatum, insular and piriform cortices are juxtaposed with vCLA embedded within layer 6 of the insular cortex and DEn within the deepest layer 3 of the piriform cortex. At this level, vCLA and DEn are continuous. Further rostrally, the emergence of the orbital cortex separates insular and piriform cortices and, therefore, vCLA from DEn. Meanwhile, vCLA and dCLA remain continuous throughout the caudo-rostral extent of the complex. Rostral to the striatum, the vCLA/dCLA complex becomes situated progressively more lateral with respect to the forceps minor of the corpus callosum.

## Conclusion

Using neuroanatomical tracing and the expression profiles of two genes that are widely accepted to be differentially expressed in the striatal claustrum, we report here that, contrary to previous reports, the rostral extent of the claustrum in the rat extends anterior to the rostral apex of the striatum. Our combined tracing and gene-marker based data represent a unified view of the position of the rostral claustrum. The functions of claustrum are a matter of continuing investigation, with cells that appear to code for aspects of extended space present in the rat claustrum (Jankowski and O’Mara, 2015) somewhat akin to the place cells and other spatial cells found in the hippocampal formation and other related areas (Grieves and Jeffery, 2017). The seeming absence of either thalamic or hippocampal inputs suggest that the spatial coding in claustrum observed by Jankowski and O’Mara (2015) is likely to be cortical in origin, perhaps originating from a combination of spatial inputs from, e.g., grid cells of the entorhinal cortex (Hafting et al., 2005), and other inputs from regions such as parieto-insular vestibular cortex (e.g., Rancz et al., 2015). The cortically encapsulated inputs and outputs of claustrum we describe here would support this proposition.

## Data Availability

The datasets generated for this study are available on request to the corresponding author.

## Ethics Statement

Animal husbandry and experimental procedures were carried out in accordance with the European Community directive, 86/609/EC, and the Cruelty to Animals Act, 1876, and were approved by the Comparative Medicine/Bioresources Ethics Committee, Trinity College, Dublin, Ireland, and followed LAST Ireland and international guidelines of good practice or, for those experiments that were performed at Cardiff University, in accordance with the United Kingdom Animals (Scientific Procedures) Act, 1986 and associated guidelines, the EU directive 2010/63/EU, as well as the Cardiff University Biological Standards Committee.

## Author Contributions

CD, MM, SO, and MJ conceived and designed the experiments. CD, MM, BF, EB, and ML performed the experiments. CD, MM, ML, and SO analyzed the data. CD and MM wrote the manuscript with contributions from SO, BF, MJ, and JA. EB and JA contributed the data or analysis tools.

## Conflict of Interest Statement

The authors declare that the research was conducted in the absence of any commercial or financial relationships that could be construed as a potential conflict of interest.

## Footnotes

1 http://mouse.brain-map.org/

2 https://imagej.net/Fiji/Downloads

## Supplementary Material

Figure S1 Photomicrographs of a sequential 1-in-4 caudo-rostral **(A–F)** series reacted against crystallin mu (Crym) ranging from a mid-striatal anterior-posterior level **(A)**, to the rostral peak of the striatum **(C)** and up to a rostral aspect of the claustrum **(F)** approximately 600 μm anterior to the striatum. Scale bar = 300 μm.

Figure S2 Images from sequential chromogenically stained crystallin mu (Crym; **A–C**) and Gng2 **(A^i^–C^i^)** sections that have been converted to 8-bit and pseudo-colored in green and red, respectively. Merged images were aligned and manually registered using landmarks (asterisks) to assess the overlap between attenuated Crym staining in the claustrum and enriched Gng2 expression. **(A–A^ii^)** shows overlap between Crym and Gng2 rostral to the striatum; **(B–B^ii^)** shows overlap at the rostral apex of the striatum and **(C–C^ii^)** show overlap at a mid-striatal anterior posterior level. In all cases, Gng2 enrichment and Crym attenuation delineated a consistent claustrum border. Scale bar = 500 μm.

Figure S3 Unilateral (right hemisphere) retrograde Tracer injections (Fluoro-gold; FG) targeting the retrosplenial cortex resulted in labeled cell soma in the claustrum both at striatal anterior-posterior (AP) levels **(C,D)** as well as rostral to the striatum **(A,B)** in a distribution that closely matched parvalbumin expression in the claustrum (See dual fluorescent label (FG and parvalbumin) from the same case in Figure 8). Scale bars = 300 μm.

Figure S4 Tracer injections of Fluorogold (FG; pseudocolored red) within retrosplenial cortex resulted in dense retrograde label throughout the extent of the ipsilateral claustrum. **(A–D)**: Schematic tracings of caudal (striatal (CPu); **(A,B)** to rostral (anterior to striatum; **C,D**) brain sections showing retrograde label in the claustrum^∗^. Rectangles in **(A–D)** show regions shown in corresponding fluorescence micrographs **(i–iii)**. Dual-fluorescence experiments showed that parvalbumin neuropil expression (PV; pseudocolored green) closely overlaid that of the FG retrograde label. Insets in **B** and **D** show anterior-posterior level relative to CPu in PV-reacted tissue. Scale bars = 200 μm.

Figure S5 Cortical **(A,B)** and thalamic **(C)** pressure injections of tracers were used to assess claustrum connectivity profiles. **(A)**, an example of a AAV-CaMKIIa-hM4D(Gi)-mCherry pressure injection into the anterior cingulate cortex (case 219#3); **(B)**, an example of a Flouro-gold pressure injection into the anterior cingulate cortex (FGRSC1) **(C)**, An example of an injection site of cholera-toxin b (red) and Fast Blue (blue) injections sites in the centromedial (CM)/paraventricular (PV)/mediodorsal (MD) and nucleus reuniens (RE)/rhomboid (Rh), respectively. Cg, anterior cingulate cortex; RSC, retrosplenial cortex; SMT, submedius thalamic nucleus. Scale bars = 200 μm.

## References

[B1] Baizer J. S. Sherwood C. C. Noonan M. Hof P. R. Mathur B. N. Innocenti G. (2014). Systems neuroscience comparative organization of the claustrum: what does structure tell us about function? *Front. Syst. Neurosci.* 8:117 10.3389/fnsys.2014.00117 PMC4079070 25071474

[B2] Buchanan K. J. Johnson J. I. (2011). Diversity of spatial relationships of the claustrum and insula in branches of the mammalian radiation. *Ann. N. Y. Acad. Sci.* 1225 E30–E63. 10.1111/j.1749-6632.2011.06022.x 21599698

[B3] Coizet V. Heilbronner S. R. Carcenac C. Mailly P. Lehman J. F. Savasta M. (2017). Organization of the anterior limb of the internal capsule in the Rat. *J. Neurosci.* 37 2539–2554. 10.1523/JNEUROSCI.3304-16.2017 28159909 PMC5354315

[B4] Dillingham C. M. Jankowski M. M. Chandra R. Frost B. E. O’Mara S. M. (2017). The claustrum: considerations regarding its anatomy, functions and a programme for research. *Brain Neurosci. Adv.* 1 1–9. 10.1177/2398212817718962 PMC7058237 32166134

[B5] Druga R. Chen S. Bentivoglio M. (1993). Parvalbumin and calbindin in the rat claustrum: an immunocytochemical study combined with retrograde tracing frontoparietal(cortex). *J. Chem. Neuroanat.* 6 399–406. 10.1016/0891-0618(93)90014-u 8142075

[B6] Erickson S. L. Melchitzky D. S. Lewis D. A. (2004). Subcortical afferents to the lateral mediodorsal thalamus in cynomolgus monkeys. *Neuroscience* 129 675–690. 10.1016/J.NEUROSCIENCE.2004.08.016 15541889

[B7] Grieves R. M. Jeffery K. J. (2017). The representation of space in the brain. *Behav. Processes* 135 113–131. 10.1016/j.beproc.2016.12.012 28034697

[B8] Hafting T. Fyhn M. Molden S. Moser M.-B. Moser E. I. (2005). Microstructure of a spatial map in the entorhinal cortex. *Nature* 436 801–806. 10.1038/nature03721 15965463

[B9] Herkenham M. (1978). The connections of the nucleus reuniens thalami: evidence for a direct thalamo-hippocampal pathway in the rat. *J. Comp. Neurol.* 177 589–609. 10.1002/cne.901770405 624792

[B10] Hinova-Palova D. V. Edelstein L. Landzhov B. V. Braak E. Malinova L. G. Minkov M. (2014a). Parvalbumin-immunoreactive neurons in the human claustrum. *Brain Struct. Funct.* 219 1813–1830. 10.1007/s00429-013-0603-x 23832597

[B11] Hinova-Palova D. V. Landzhov B. Dzhambazova E. Minkov M. Edelstein L. Malinova L. (2014b). Neuropeptide Y immunoreactivity in the cat claustrum: a light- and electron-microscopic investigation. *J. Chem. Neuroanat.* 61–62, 107–119. 10.1016/j.jchemneu.2014.08.007 25157673

[B12] Jankowski M. M. O’Mara S. M. (2015). Dynamics of place, boundary and object encoding in rat anterior claustrum. *Front. Behav. Neurosci.* 9:250. 10.3389/fnbeh.2015.00250 26557060 PMC4617374

[B13] Kim J. Matney C. J. Roth R. H. Brown S. P. (2016). Synaptic organization of the neuronal circuits of the claustrum. *J. Neurosci.* 36 773–784. 10.1523/JNEUROSCI.3643-15.2016 26791208 PMC4719014

[B14] Kitanishi T. Matsuo N. (2016). Organization of the claustrum-to-entorhinal cortical connection in mice. *J. Neurosci.* 37 269–280. 10.1523/JNEUROSCI.1360-16.2016 28077707 PMC6596572

[B15] Mathiasen M. L. Amin E. Nelson A. J. D. Dillingham C. M. O’Mara S. M. Aggleton J. P. (2019). Separate cortical and hippocampal cell populations target the rat nucleus reuniens and mammillary bodies. *Eur. J. Neurosci.* 10.1111/ejn.14341 [Epub ahead of print]. PMIDPMID:30633830 30633830 PMC6618334

[B16] Mathur B. N. (2014). The claustrum in review. *Front. Syst. Neurosci.* 8:48 10.3389/fnsys.2014.00048 PMC3983483 24772070

[B17] Mathur B. N. Caprioli R. M. Deutch A. Y. (2009). Proteomic analysis illuminates a novel structural definition of the claustrum and insula. *Cereb. Cortex* 19 2372–2379. 10.1093/cercor/bhn253 19168664 PMC2742595

[B18] McKenna J. T. Vertes R. P. (2004). Afferent projections to nucleus reuniens of the thalamus. *J. Comp. Neurol.* 480 115–142. 10.1002/cne.20342 15514932

[B19] Patzke N. Innocenti G. M. Manger P. R. (2014). The claustrum of the ferret: afferent and efferent connections to lower and higher order visual cortical areas. *Front. Syst. Neurosci.* 8:31. 10.3389/fnsys.2014.00031 24616671 PMC3937871

[B20] Paxinos G. Watson C. (2005). *The Rat Brain in Stereotaxic Coordinates.* San Diego: Elsevier Academic Press.

[B21] Pirone A. Cozzi B. Edelstein L. Peruffo A. Lenzi C. Quilici F. (2012). Topography of Gng2- and netring2-expression suggests an insular origin of the human claustrum. *PLoS One* 7:e44745. 10.1371/journal.pone.0044745 22957104 PMC3434180

[B22] Pirone A. Magliaro C. Giannessi E. Ahluwalia A. (2015). Parvalbumin expression in the claustrum of the adult dog. an immunohistochemical and topographical study with comparative notes on the structure of the nucleus. *J. Chem. Neuroanat.* 6 33–42. 10.1016/j.jchemneu.2015.02.004 25724540

[B23] Puelles L. Ayad A. Alonso A. Sandoval J. E. MartÍnez-de-la-Torre M. Medina L. (2016). Selective early expression of the orphan nuclear receptor Nr4a2 identifies the claustrum homolog in the avian mesopallium: impact on sauropsidian/mammalian pallium comparisons. *J. Comp. Neurol.* 524 665–703. 10.1002/cne.23902 26400616

[B24] Qadir H. Krimmel S. R. Mu C. Poulopoulos A. Seminowicz D. A. Mathur B. N. (2018). Structural connectivity of the anterior cingulate cortex. claustrum, and the anterior insula of the Mouse. *Front. Neuroanat.* 12:100. 10.3389/fnana.2018.00100 30534060 PMC6276828

[B25] Rahman F. E. Baizer J. S. (2007). Neurochemically defined cell types in the claustrum of the cat. *Brain Res.* 1159 94–111. 10.1016/j.brainres.2007.05.011 17582386

[B26] Rancz E. A. Moya J. Drawitsch F. Brichta A. M. Canals S. Margrie T. W. (2015). Widespread vestibular activation of the rodent cortex. *J. Neurosci.* 35 5926–5934. 10.1523/JNEUROSCI.1869-14.2015 25878265 PMC4397593

[B27] Remedios R. Logothetis N. K. Kayser C. (2010). Unimodal responses prevail within the multisensory claustrum. *J. Neurosci.* 30 12902–12907. 10.1523/JNEUROSCI.2937-10.2010 20881109 PMC6633510

[B28] Smith J. B. Alloway K. D. (2010). Functional specificity of claustrum connections in the rat: interhemispheric communication between specific parts of motor cortex. *J. Neurosci.* 30 16832–16844. 10.1523/JNEUROSCI.4438-10.2010 21159954 PMC3010244

[B29] Smith J. B. Alloway K. D. Hof P. R. Orman R. Reser D. H. Watakabe A. (2018). The relationship between the claustrum and endopiriform nucleus: a perspective towards consensus on cross-species homology. *J. Comp. Neurol.* 10.1002/cne.24537 [Epub ahead of print]. 30225888 PMC6421118

[B30] Smith J. B. Alloway K. D. Mathur B. N. Garraghty P. E. Sherk H. (2014). Interhemispheric claustral circuits coordinate sensory and motor cortical areas that regulate exploratory behaviors. *Front. Syst. Neurosci.* 8:93. 10.3389/fnsys.2014.00093 24904315 PMC4032913

[B31] Smythies J. R. Edelstein L. R. Ramachandran V. S. (2014). Hypotheses relating to the function of the claustrum. claustrum struct. funct. *Clin. Neurosci.* 6 299–352. 10.3389/fnint.2012.00053

[B32] Van De Werd H. J. J. M. Uylings H. B. M. (2008). The rat orbital and agranular insular prefrontal cortical areas: a cytoarchitectonic and chemoarchitectonic study. *Brain Struct. Funct.* 212 387–401. 10.1007/s00429-007-0164-y 18183420

[B33] Vertes R. P. Hoover W. B. Do Valle A. C. Sherman A. Rodriguez J. J. (2006). Efferent projections of reuniens and rhomboid nuclei of the thalamus in the rat. *J. Comp. Neurol.* 499 768–796. 10.1002/cne.21135 17048232

[B34] Vertes R. P. Hoover W. B. Rodriguez J. J. (2012). Projections of the central medial nucleus of the thalamus in the rat: node in cortical, striatal and limbic forebrain circuitry. *Neuroscience* 219 120–136. 10.1016/J.NEUROSCIENCE.2012.04.067 22575585

[B35] Wang Q. Ng L. Harris J. A. Feng D. Li Y. Royall J. J. (2017). Organization of the connections between claustrum and cortex in the mouse. *J. Comp. Neurol.* 525 1317–1346. 10.1002/cne.24047 27223051 PMC5324679

[B36] Watakabe A. (2017). In situ hybridization analyses of claustrum-enriched genes in marmosets. *J. Comp. Neurol.* 525 1442–1458. 10.1002/cne.24021 27098836

[B37] White M. G. Cody P. A. Bubser M. Wang H.-D. Deutch A. Y. Mathur B. N. (2017). Cortical hierarchy governs rat claustrocortical circuit organization. *J. Comp. Neurol.* 525 1347–1362. 10.1002/cne.23970 26801010 PMC4958609

[B38] Yoshida K. McCormack S. España R. A. Crocker A. Scammell T. E. (2005). Afferents to the orexin neurons of the rat brain. *J. Comp. Neurol.* 494 845–861. 10.1002/cne.20859 16374809 PMC2259441

[B39] Zhang X. Hannesson D. K. Saucier D. M. Wallace A. E. Howland J. Corcoran M. E. (2001). Susceptibility to kindling and neuronal connections of the anterior claustrum. *J. Neurosci.* 21 3674–3687. 10.1523/jneurosci.21-10-03674.2001 11331397 PMC6762482

[B40] Zingg B. Dong H.-W. Tao H. W. Zhang L. I. (2018). Input-output organization of the mouse claustrum. *J. Comp. Neurol.* 526 2428–2443. 10.1002/cne.24502 30252130 PMC6196111

