# Supplementary figures and images for: The Anatomical Boundary of the Rat Claustrum
Source: Front Neuroanat. 2019 May 31;13:53. doi: 10.3389/fnana.2019.00053 (PMC6555083; doi:10.3389/fnana.2019.00053)

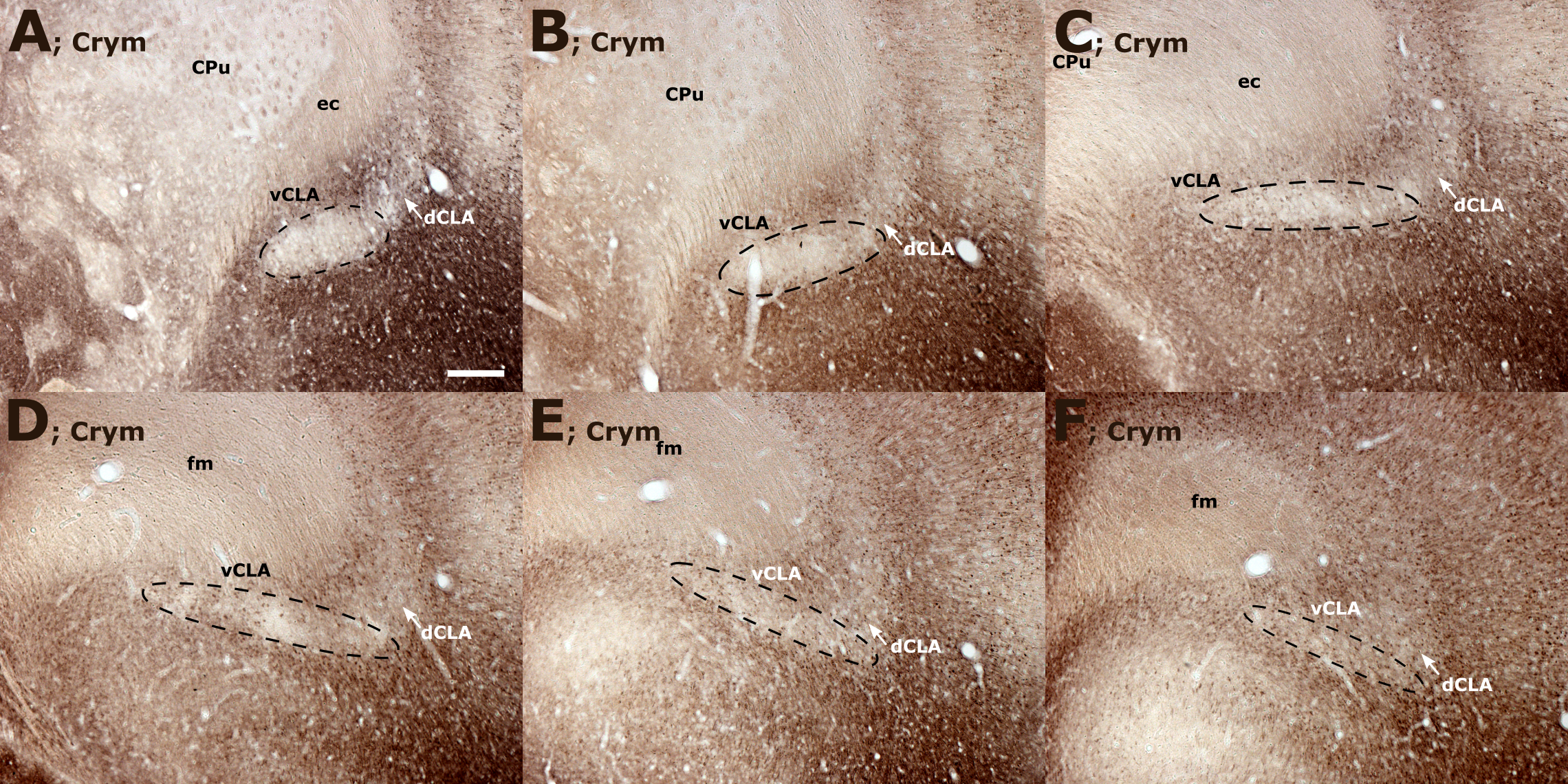

Supplement: Figure S1 — Photomicrographs of a sequential 1-in-4 caudo-rostral (A–F) series reacted against crystallin mu (Crym) ranging from a mid-striatal anterior-posterior level (A), to the rostral peak of the striatum (C) and up to a rostral aspect of the claustrum (F) approximately 600 μm anterior to the striatum. Scale bar = 300 μm. [file Image_1.TIF]

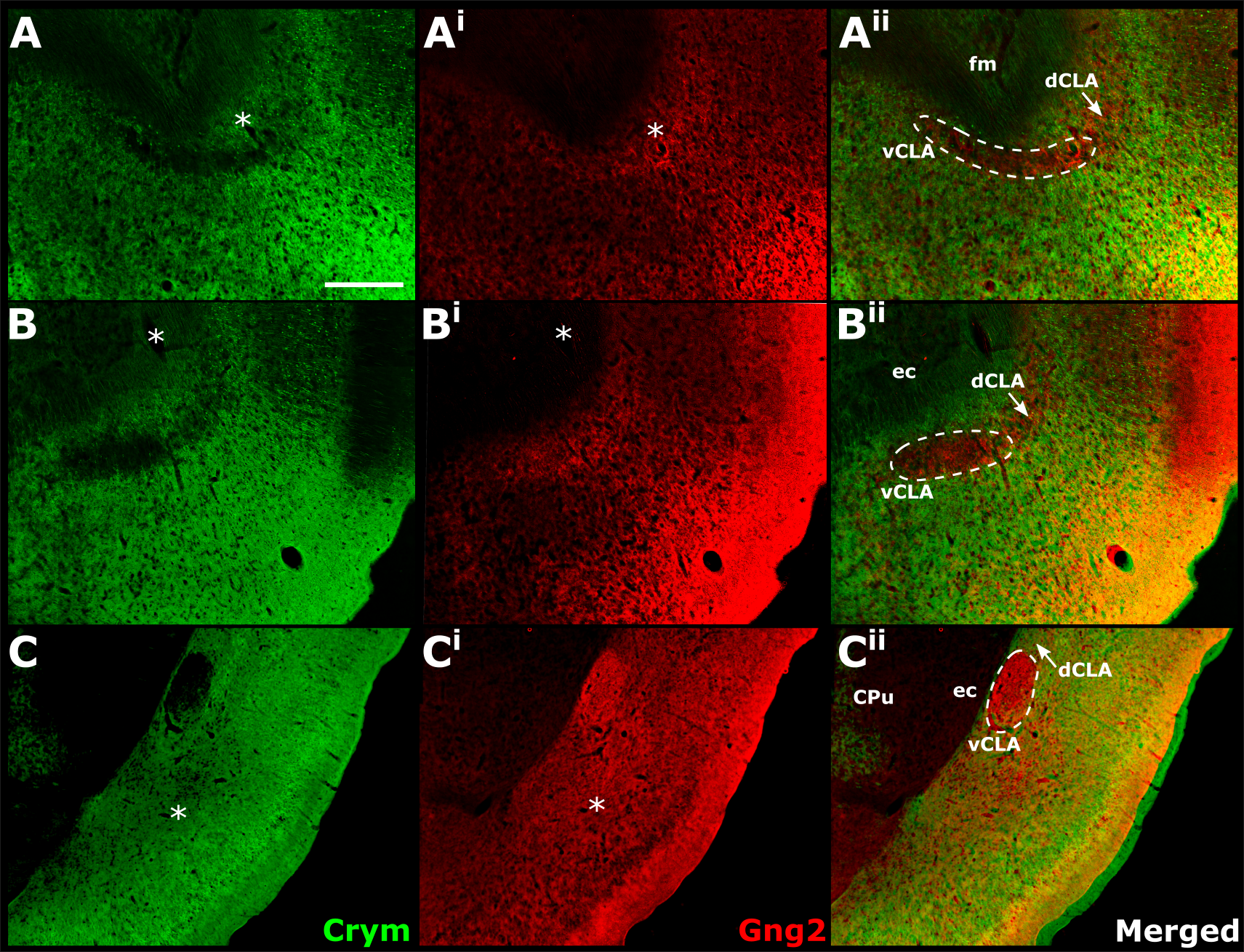

Supplement: Figure S2 — Images from sequential chromogenically stained crystallin mu (Crym; A–C) and Gng2 (Ai–Ci) sections that have been converted to 8-bit and pseudo-colored in green and red, respectively. Merged images were aligned and manually registered using landmarks (asterisks) to assess the overlap between attenuated Crym staining in the claustrum and enriched Gng2 expression. (A–Aii) shows overlap between Crym and Gng2 rostral to the striatum; (B–Bii) shows overlap at the rostral apex of the striatum and (C–Cii) show overlap at a mid-striatal anterior posterior level. In all cases, Gng2 enrichment and Crym attenuation delineated a consistent claustrum border. Scale bar = 500 μm. [file Image_2.TIF]

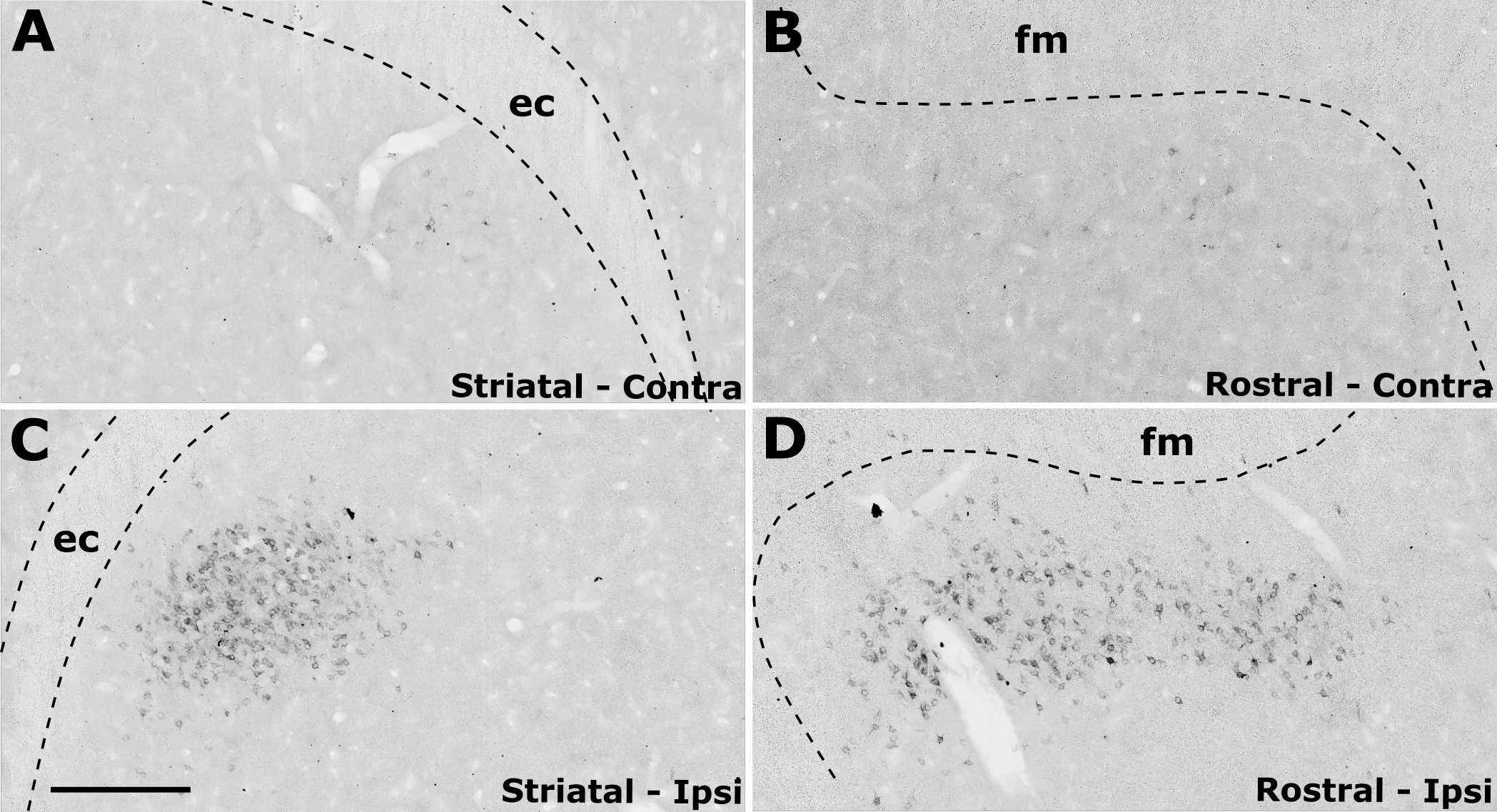

Supplement: Figure S3 — Unilateral (right hemisphere) retrograde Tracer injections (Fluoro-gold; FG) targeting the retrosplenial cortex resulted in labeled cell soma in the claustrum both at striatal anterior-posterior (AP) levels (C,D) as well as rostral to the striatum (A,B) in a distribution that closely matched parvalbumin expression in the claustrum (See dual fluorescent label (FG and parvalbumin) from the same case in Figure 8). Scale bars = 300 μm. [file Image_3.TIF]

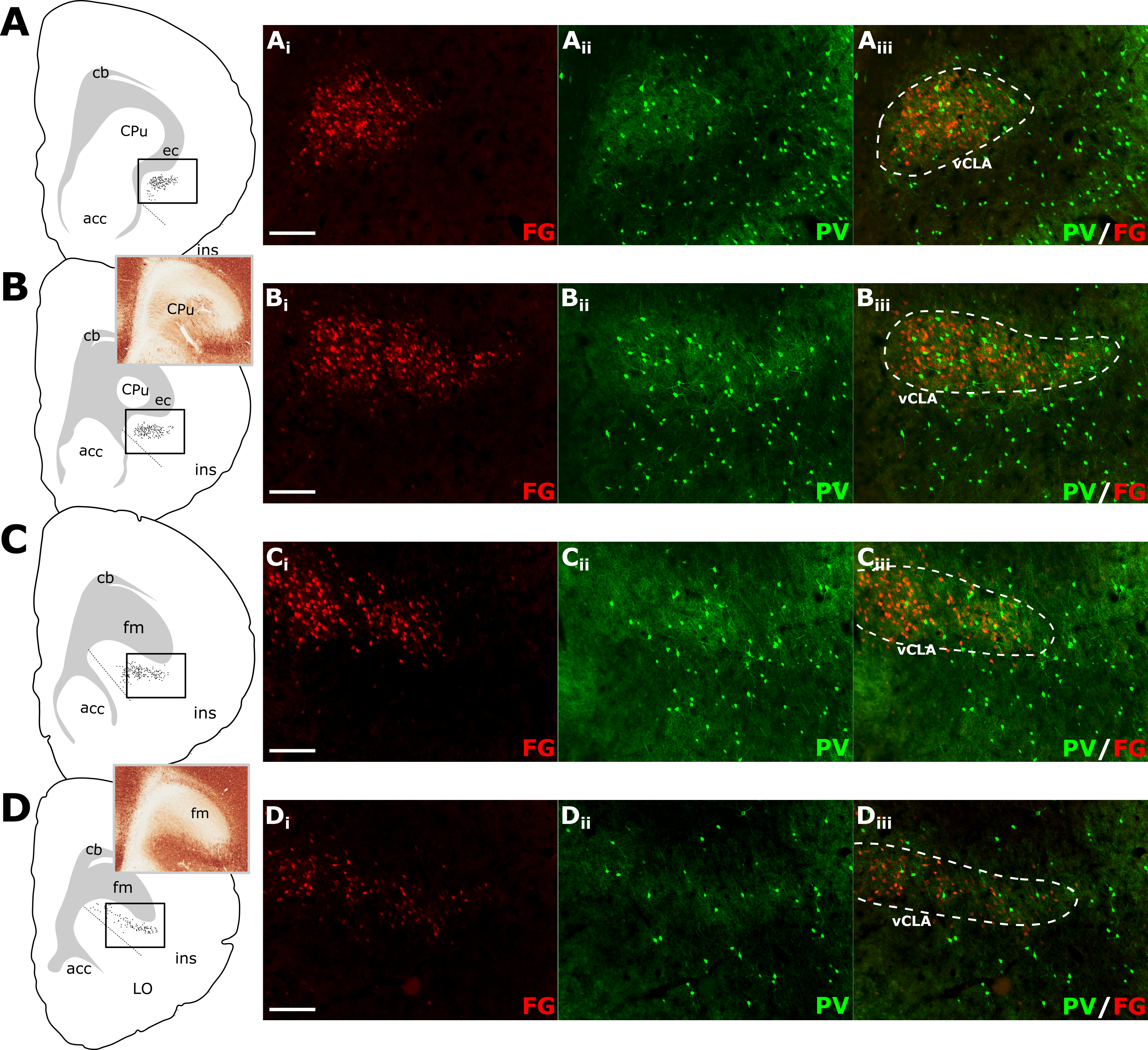

Supplement: Figure S4 — Tracer injections of Fluorogold (FG; pseudocolored red) within retrosplenial cortex resulted in dense retrograde label throughout the extent of the ipsilateral claustrum. (A–D): Schematic tracings of caudal (striatal (CPu); (A,B) to rostral (anterior to striatum; C,D) brain sections showing retrograde label in the claustrum∗. Rectangles in (A–D) show regions shown in corresponding fluorescence micrographs (i–iii). Dual-fluorescence experiments showed that parvalbumin neuropil expression (PV; pseudocolored green) closely overlaid that of the FG retrograde label. Insets in B and D show anterior-posterior level relative to CPu in PV-reacted tissue. Scale bars = 200 μm. [file Image_4.TIF]

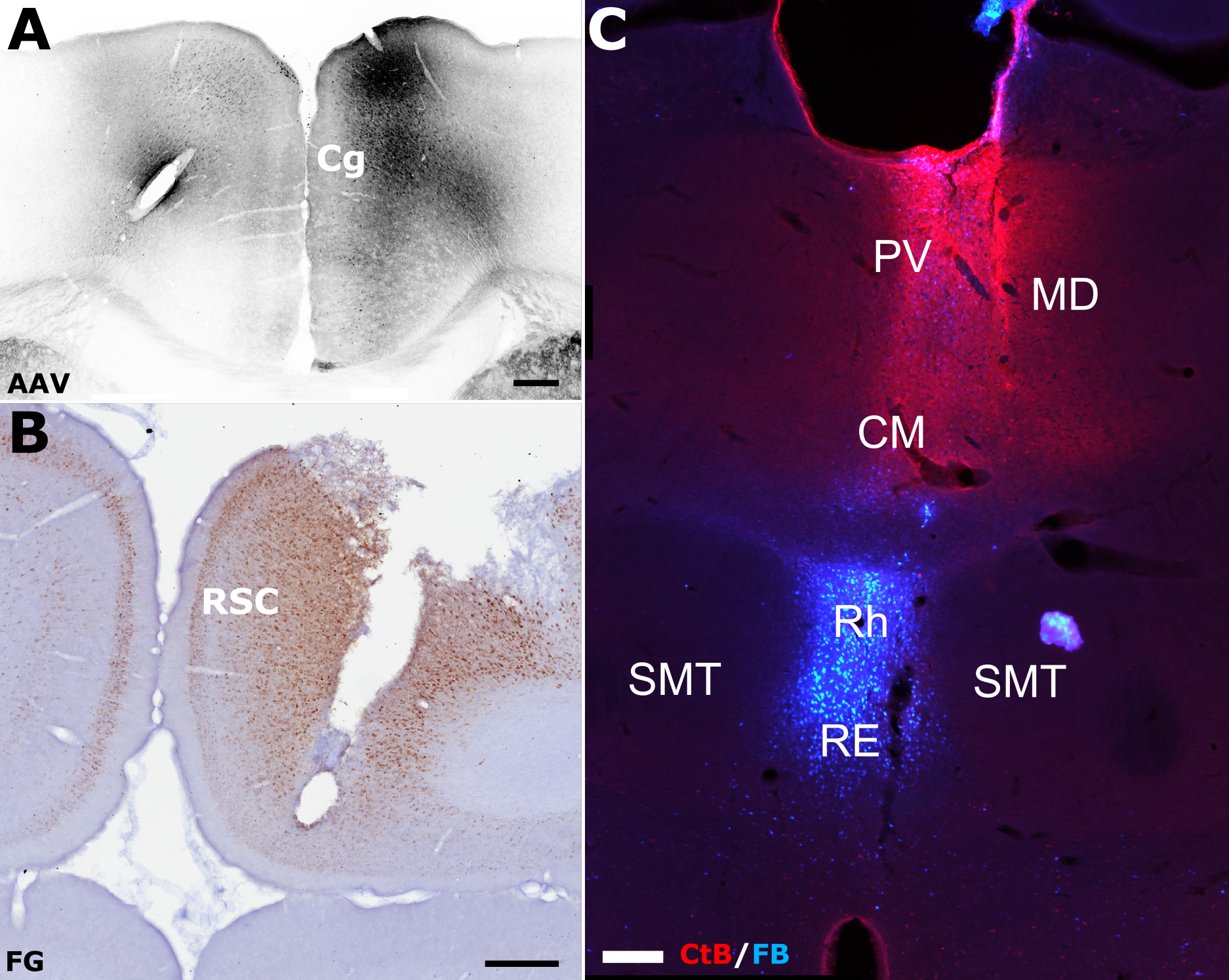

Supplement: Figure S5 — Cortical (A,B) and thalamic (C) pressure injections of tracers were used to assess claustrum connectivity profiles. (A), an example of a AAV-CaMKIIa-hM4D(Gi)-mCherry pressure injection into the anterior cingulate cortex (case 219#3); (B), an example of a Flouro-gold pressure injection into the anterior cingulate cortex (FGRSC1) (C), An example of an injection site of cholera-toxin b (red) and Fast Blue (blue) injections sites in the centromedial (CM)/paraventricular (PV)/mediodorsal (MD) and nucleus reuniens (RE)/rhomboid (Rh), respectively. Cg, anterior cingulate cortex; RSC, retrosplenial cortex; SMT, submedius thalamic nucleus. Scale bars = 200 μm. [file Image_5.TIF]
